# Expression of glutamate carboxypeptidase II in the glial tumor recurrence evaluated in vivo using radionuclide imaging

**DOI:** 10.1038/s41598-021-04613-w

**Published:** 2022-01-13

**Authors:** Jolanta Kunikowska, Rafał Czepczyński, Dariusz Pawlak, Henryk Koziara, Kacper Pełka, Leszek Królicki

**Affiliations:** 1 Nuclear Medicine Department, Medical University of Warsaw, Warsaw, Poland; 2 Department of Endocrinology, Metabolism and Internal Diseases, Poznan University of Medical Sciences, Przybyszewskiego 49, 60-355 Poznań, Poland; 3 Radioisotope Centre POLATOM, National Centre for Nuclear Research, Otwock, Poland; 4 Department of Neurosurgery, Maria Skłodowska-Curie National Research Institute of Oncology, Warsaw, Poland; 5 Department of Methodology, Laboratory of Centre for Preclinical Research, Medical University of Warsaw, Warsaw, Poland

**Keywords:** Biomarkers, Molecular medicine, Neurology, Oncology

## Abstract

Glutamate carboxypeptidase II (GCP), also known as prostate specific membrane antigen (PSMA) has been found to be expressed in glioma vasculature in in-vitro studies. GCP expression can be traced with the use of [^68^Ga]Ga-PSMA-11 PET/CT used routinely for prostate cancer imaging. The aim of this paper was to analyze GCP expression in the recurrent glial tumors in vivo. 34 patients (pts.) aged 44.5 ± 10.3 years with suspicion of recurrence of histologically confirmed glioma grade III (6 pts.) and grade IV (28 pts.) were included in the study. All patients underwent contrast-enhanced MR and [^68^Ga]Ga-PSMA-11 PET/CT. No radiopharmaceutical-related adverse events were noted. PET/CT was positive in all the areas suspected for recurrence at MR in all the patients. The recurrence was confirmed by histopathological examinations or follow-up imaging in all cases. The images showed a very low background activity of the normal brain. Median maximal standard uptake value (SUV_max_) of the tumors was 6.5 (range 0.9–15.6) and mean standard uptake value (SUV_mean_) was 3.5 (range 0.9–7.5). Target-to-background (TBR) ratios varied between 15 and 1400 with a median of 152. Target-to-liver background ratios (TLR) ranged from 0.2 to 2.6, the median TLR was 1.3. No significant difference of the measured parameters was found between the subgroups according to the glioma grade. High GCP expression in the recurrent glioma was demonstrated in-vivo with the use of [^68^Ga]Ga-PSMA-11 PET/CT. As the treatment options in recurrent glioma are limited, this observation may open new therapeutic perspectives with the use of radiolabeled agents targeting the GCP.

## Introduction

Primary glial tumors are the most prevalent types of brain tumors in adults, with heterogenous histology. The World Health Organization Classification of Tumors of the Central Nervous System (WHO 2016) is based not only on histopathological features, but also on the expression of specific molecular biomarkers^1^. According to this classification, different histopathological subtypes, of which astrocytoma (60–70%), oligodendroglioma (10–30%) and ependymoma (< 10%) are the most common, are stratified into four prognostic grades.

Glutamate carboxypeptidase II (GCP), also known as prostate specific membrane antigen (PSMA) is overexpressed in prostate cancer cells. Immunohistochemical examinations revealed the expression of PSMA not only in the prostate cancer but also in other tumors, including glial tumors, though with various intensity^2,3^. Its physiological role includes the *N*-acetyl-aspartyl-glutamate (NAAG)-hydrolysing activity in the nervous system^4,5^. As a carboxypeptidase. GCP is responsible for the glutamate release from NAAG. The NAAG was primary known as a precursor of a neurotransmitter, which is highly expressed in neuronal synapses^4,6^. Further studies correlated NAAG level with a progression of neurological diseases, such as Alzheimer, Huntington disease or schizophrenia^7^.

Recent investigations by Tanjore Ramanthan et al. showed GCP expression in the proliferating microvasculature (PM) of glioblastomas. Moreover, only the vascular PSMA expression in gliomas was associated with poor overall survival^8^

Distribution of GCP expression can be traced in vivo using radiopharmaceuticals specifically targeting the GCP molecules in the cellular membranes. The most common radiotracer is [^68^Ga]Ga-PSMA-11. For several years, [^68^Ga]Ga-PSMA-11 has been successfully used to analyse distribution of the cancer cells showing elevated expression of GCP, mainly in prostate cancer. As a highly sensitive and cancer-specific method, the PET/CT with [^68^Ga]Ga-PSMA-11 has become a routine tool for the imaging of prostate cancer, for both, staging and detection of recurrence^9^. In case of gliomas, several studies have focused on the use of [^68^Ga]Ga-PSMA-11 PET/CT in patients with glioblastoma multiforme and (even less) in other types of glial tumors^10–16^.

The aim of this paper was to analyze GCP expression in the recurrent glial tumors with the use of [^68^Ga]Ga-PSMA-11 PET/CT.

## Materials and methods

This was a single-institution study performed in accordance with the Declaration of Helsinki. It was approved by the Ethical Committee of Medical University of Warsaw (KB/235/2016 and KB/2/A/2018). Written informed consent was obtained from all patients.

### Patients

Patients with a suspicion of recurrence of glial tumors based on MR evaluation were qualified to the study. The study group consisted of 34 patients (22 male, 12 female pts.) aged 44.5 ± 10.3 years. The histopathological diagnosis was obtained before treatment initiation. 6 patients were diagnosed of grade III glioma and 28 patients of grade IV (glioblastoma). The detailed WHO 2016 classification and patient’s treatment history of each patient is listed in Table 1.

**Table 1:** Detailed patients’ data.

No	Gender	Age	Grade and WHO 2016 type	Localisation (lobe)	Treatment	Other treatment	Time to recurrence (months)	Recurrent tumor volume (cm^3^)
1	M	35	III *Anaplastic astrocytoma NOS*	R frontal	S		3.5	23.8
2	M	39	III *Anaplastic astrocytoma IDH wildtype*	R parieto-occipital	S, RTx, CHT	[^213^Bi]Bi-DOTA-SP	37.3	28.4
3	F	49	III *Anaplastic astrocytoma IDH wildtype*	R temporalo-occipital	S, RTx, CHT		108.4	108.0
4	F	43	III *Oligodendroglioma IDH mutant*	R frontal multifocal	S, RTx	Irinotecan	87.1	49.7
5	K	27	III *Anaplastic astrocytoma NOS*	Multifocal	S, RTx, CHT	[^225^Ac]Ac-DOTA-SP TMZ	59.0	73.3
6	M	50	III *Secondary Anaplastic oligodendroglioma NOS*	L frontal multifocal	S,RTx,CHT	Irinotecan	200.0	18.5
7	M	69	IV *Glioblastoma IDH wildtype*	R parietal	S, Rtx	TMZ	3.9	22.9
8	M	54	IV *Glioblastoma NOS*	R frontal	S, RTx	TMZ	28.2	32.6
9	M	45	IV *Glioblastoma NOS*	L deep structures	S, RTx	TMZ	11.0	42.3
10	F	44	IV *Secondary Glioblastoma IDH wildtype*	L temporal	S, RTx, CHT	[^213^Bi]Bi-DOTA-SP	184.1	22.1
11	M	33	IV *Glioblastoma IDH mutant*	R fronto-parietal	S, RTx, CHT		15.9	25.3
12	F	51	IV *Glioblastoma IDH wildtype—giant cell*	R fronto-parietal	S, RTx, CHT	[^213^Bi]Bi-DOTA-SP	46.7	107.2
13	M	16	IV *Glioblastoma IDH mutant*	L fronto-parietal	S, RTx, CHT		3.2	244.2
14	M	37	IV *Secondary Glioblastoma NOS*	R frontal	S, RTx, CHT	TMZ	111.5	70.6
15	F	25	IV *Secondary Glioblastoma IDH mutant*	L insular	S, RTx, CHT	[^225^Ac]Ac-DOTAGA-SP	64.6	4.7
16	M	47	IV *Glioblastoma NOS*	L parieto-occipital	S, RTx, CHT		6.3	120.2
17	M	43	IV *Secondary Glioblastoma NOS*	L parietal	S, RTx	TMZ	49.1	6.3
18	F	47	IV *Glioblastoma NOS*	R parietal	S, RTx, CHT		19.4	113.5
19	M	42	IV *Glioblastoma IDH mutant*	R fronto-parietal	S, RTx, CHT	TMZ	27.2	142.2
20	F	48	IV *Glioblastoma IDH wildtype*	R temporal	S, RTx, CHT		11.2	40.8
21	M	44	IV *Glioblastoma IDH wildtype*	L fronto-temporalo-parietal	S, RTx, CHT	Irinotecan	19.8	51.4
22	F	54	IV *Glioblastoma IDH wildtype*	L parieto-occipital	S, RTx, CHT		5.3	22.1
23	M	55	IV *Glioblastoma NOS*	Multifocal	S, RTx, CHT		7.5	29.1
24	F	41	IV *Secondary Glioblastoma IDH mutant*	R corpus callosum	S, RTx, CHT	TMZ	29.8	8.4
25	M	44	IV *Secondary Glioblastoma IDH mutant*	R frontal	S, RTx	TMZ	54.3	94.9
26	F	42	IV *Secondary Glioblastoma IDH wildtype*	R parietal	S, RTx, CHT		119.2	40.3
27	M	38	IV *Glioblastoma NOS*	R parieto-occipital	S, RTx, CHT	TMZ	10.9	16.5
28	M	37	IV *Glioblastoma IDH mutant*	R parietal	S, RTx, CHT	Gene therapy	32.7	56.0
29	M	64	IV *Glioblastoma IDH wildtype*	Multifocal	S,RTx, CHT		2.0	41.6
30	F	53	IV *Glioblastoma IDH mutant*	R parieto-temporal multifocal	S, RTx, CHT	Gamma knife	44.8	38.9
31	M	53	IV *Glioblastoma IDH wildtype*	Multifocal	S, CHT	TMZ	4.0	11.4
32	M	50	IV *Glioblastoma NOS*	L frontal	S, RTx		6.3	52.7
33	M	39	IV *Glioblastoma IDH wildtype*	R frontal	S, RTx, CHT	Irinotecan	13.3	77.0
34	M	46	IV *Glioblastoma IDH wildtype*	L fronto-parietal	S, RTx, CHT		10.1	200.2

*R* right, *L* left, *S* surgery, *RTx* radiotherapy, *CHT* chemotherapy, *TMZ* temozolamide.

All patients underwent contrast-enhanced MR and [^68^Ga]Ga-PSMA-11 PET/CT within a 2 weeks’ interval.

### MR

MR examinations were performed using GE Excite HD 1.5 T (GE Healthcare, USA) or Siemens 3 T MR scanner (Siemens Medical Solutions Inc, USA).

As recommended by Ellingson et al.^17^, MR protocol included: T1WI, FLAIR, DWI and after gadolinium-based contrast administration: axial bi-dimensional T2WI and three-dimensional T1WI. The images were viewed and interpreted by two experienced radiologists.

According to the adopted criteria, it was assumed that in patients with high grade gliomas the recurrence was characterized mainly by contrast enhancement in T1WI images and hyperintense lesions in T2WI and FLAIR. In non-enhancing, low grade gliomas recurrence was characterized by hyperintense lesions in T2WI and FLAIR. DWI sequence was used for differentiation between recurrence and pseudoprogression.

### [^68^Ga]Ga-PSMA-11 PET/CT

PSMA-11 kit containing 20 µg of PSMA-11 (Glu-CO-Lys(Ahx)-HBED-CC) and 60 mg of sodium acetate (POLATOM, Poland) and eluates from a ^68^Ge/^68^Ga Galliapharm generator (Eckert & Ziegler, Germany) were used for the [^68^Ga]Ga-PSMA-11 preparation. The radiopharmaceutical labeling of [^68^Ga]Ga-PSMA-11 was performed as previously described^18^.

The PET/CT image acquisition was performed 60 min post injection of [^68^Ga]Ga-PSMA-11 (2 MBq per kg body weight) from the skull to the mid-thigh (3-min per bed position, three iterations, 21 subsets) on a Biograph 64 TruePoint (Siemens Medical Solutions Inc., USA).

Image analysis was performed using the Siemens Workstation (Syngovia, MMWS, Siemens Medical Solutions Inc, USA). PET/CT scans were analyzed by two certified nuclear medicine physicians with more than 5 years’ experience in PET imaging.

On visual evaluation, any cerebral focal uptake higher than the background was interpreted as a positive lesion. For quantitative analysis, the maximal standardized uptake value (SUV_max_) and mean standardized uptake value (SUV_mean_) of each positive lesion were measured using spherical volume of interest. Target-to-background ratios (TBR) were calculated using SUV_max_ of the lesion divided by SUV_max_ of the background that was measured using a volume of interest of a similar diameter, placed in an unaffected region. Target-to-liver ratios (TLR) were calculated by dividing SUV_max_ of the lesion by SUV_mean_ of the liver (a similar volume in the center of the right liver lobe). The recurrent tumor volume was measured using the SUV_max_ threshold of 10% as previously described^19^. SUV_mean_ was measured in the recurrent tumor volume.

### Statistical methods

In order to summarize the patients’ characteristics, means and standard deviations, medians and range depending on the parameters' distribution were used.

Calculations were done on GraphPad PRISM 5 (GraphPad Software Inc) and Excel for MAC (version 16.28, 2019 Microsoft). A non-parametric ANOVA test was used for the statistical comparison between SUV_max_, SUV_mean_, TBR and TLR.

## Results

### Toxicity and safety

The injection of [^68^Ga]Ga-PSMA-11 was well tolerated and no adverse events were recorded.

### MR findings

In all patients with recurrence of glioblastoma and anaplastic astrocytoma, focal parenchymal swelling, hyperintense on T2WI and FLAIR with low signal on T1WI were found. Peripheral irregular ring-shaped enhancement around the postoperative cavity or necrosis on post-contrast T1WI were observed. Increase of rCBV in the same regions was visible, as well.

In patients with oligodendroglioma, the signal intensity was lower in comparison to the grey matter on T1WI images and was hyperintense and heterogenous on T2WI/FLAIR images. No symptoms of peritumoral edema were observed. Contrast enhancement was seen in the case of oligodendroglioma grade III.

### [^68^Ga]Ga-PSMA-11 PET/CT image analysis

Physiological uptake in lacrimal and salivary glands, tonsils, liver, spleen, kidneys and duodenum was observed in all the patients. The background normal brain tissue showed extremely low uptake in all cases, with SUV_max_ < 0.23 in all and < 0.1 in the majority of images.

In all the lesions detected at MR, an increased accumulation of [^68^Ga]Ga-PSMA-11 was found, though with different intensity. In seven patients, multiple foci of increased [^68^Ga]Ga-PSMA-11 accumulation were visualized: two lesions in three patients, three lesions in two, and four lesions in two patients. In these subjects, a lesion with the highest uptake was selected for the per-patient analysis. Median SUV_max_ in the entire group was 6.5 (range 0.9–15.6) and SUV_mean_ was 3.5 (range 0.9–7.5). TBR varied between 15 and 1400 with a median of 152. TLR ranged from 0.2 to 2.6, the median TLR was 1.3.

These values were assessed separately in subgroups according to the glioma grade III–IV (see Table 2). No statistically significant difference was found between the subgroups in any of the analyzed parameters.

**Table 2:** Analyzed parameters: SUV_max_, SUV_mean_, TBR and TLR in the subgroups—per-patient analysis.

Grade	III		IV	
Number of pts	6		28	
	Range	Median	Range	Median
SUV_max_	2.1–11.2	6.2	1.3–15.6	7.1
SUV_mean_	2.1–5.5	3.3	1.3–7.5	3.5
TBR	21–186	72	18–1400	89
TLR	0.3–2.6	0.8	0.7–2.8	1.1

Subsequently, per-lesion analysis was performed in which 47 foci found in 34 patients (27 pts. with unifocal and 7 with multifocal recurrence) were inspected separately (Table 3). No significant difference between the groups in any parameter was found either.

**Table 3:** Analysed parameters: SUV_max_, SUV_mean_, TBR and TLR in the subgroups—per-lesion analysis.

Grade	III		IV	
No. of lesions	11		36	
	Range	Median	Range	Median
SUV_max_	0.9–11.2	5.6	1.3–15.6	6.6
SUV_mean_	0.9–5.5	3.3	1.3–7.5	3.5
TBR	15–186	70	18–1400	82
TLR	0.2–2.6	0.8	0.4–2.8	1.2

**Figure 1:**
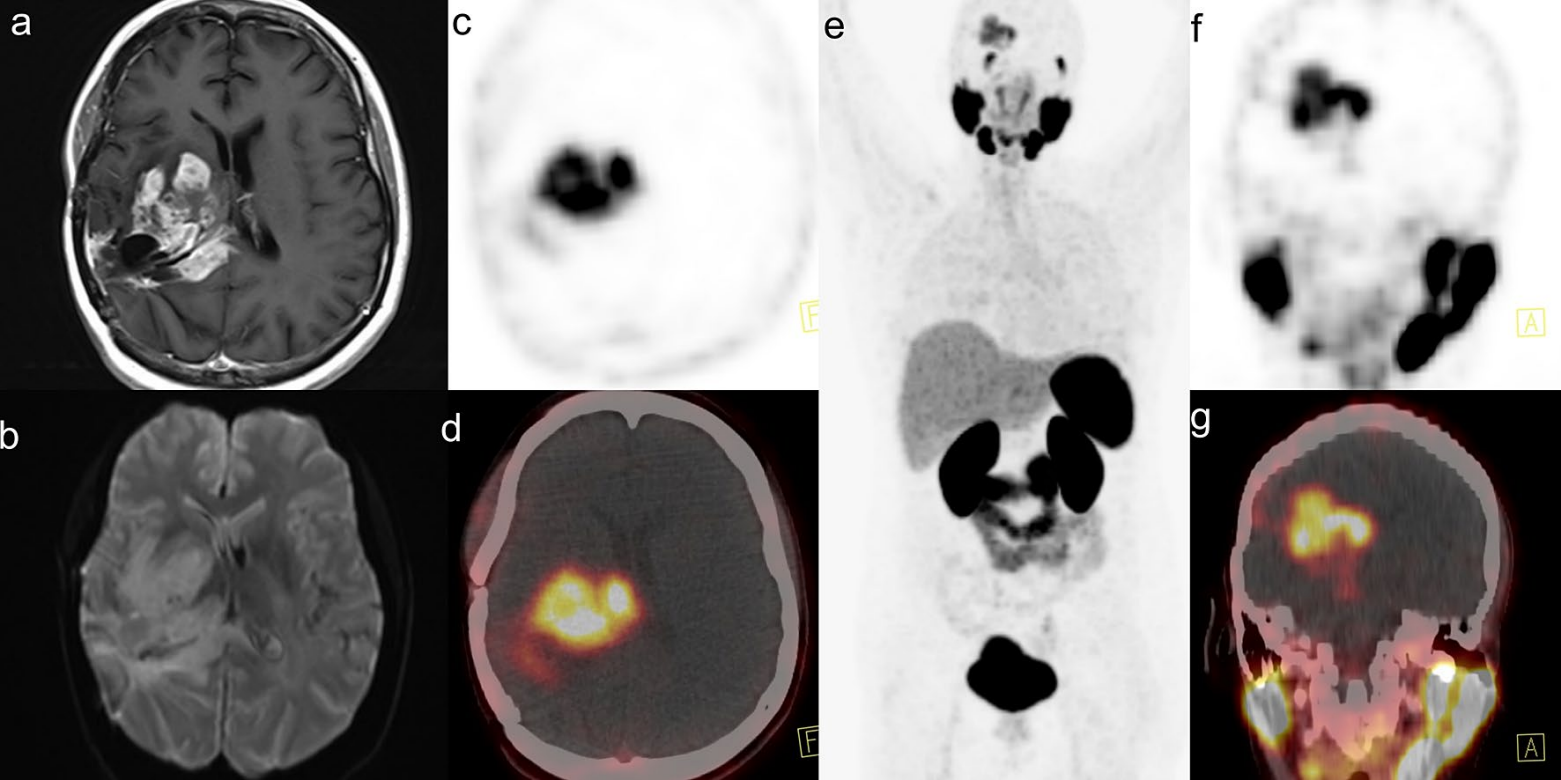
Male, 39 years, with a recurrent anaplastic astrocytoma IDH wildtype grade III in the right parieto-occipital lobe. Three years after surgery, and 15 months after local radionuclide therapy with [^213^Bi]Bi-DOTA-SP, a contrast-enhanced mass around the surgical cavity was detected with MR. [^68^Ga]Ga-PSMA-11 accumulation in the tumor was found with the SUV_max_ 4.9, SUV_mean_ 3.3, TBR 70 and TLR 0.8 (**a**—contrast-enhanced T1WI, **b**—DWI, **c**—PET transverse image, **d**—fused PET/CT transverse image, **e**—maximal intensity projection, **f**—PET sagittal image, **g**—fused PET/CT sagittal image).

**Figure 2:**
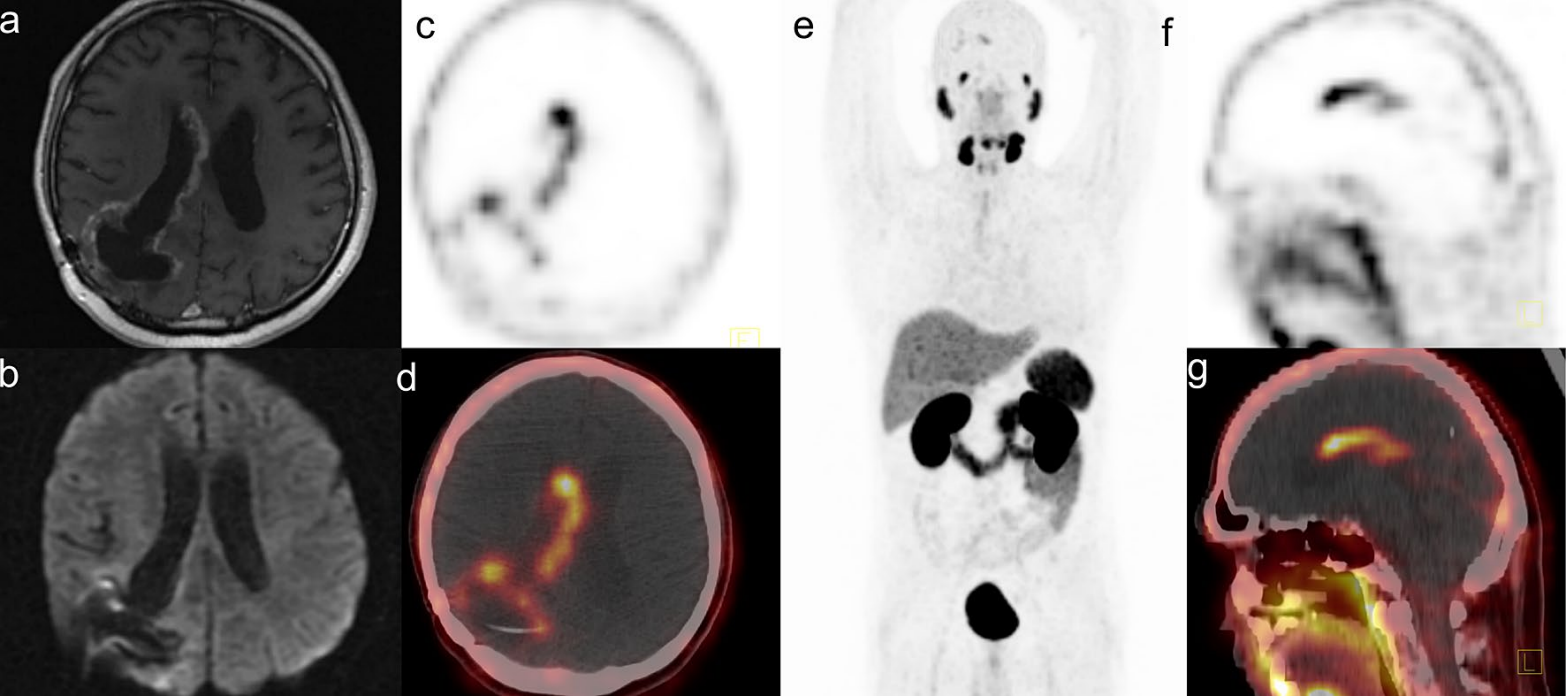
Female, 42 years, with a recurrent secondary glioblastoma in the right parietal lobe. Initially, she was diagnosed with glioma tumor grade II and underwent surgery. A follow-up MR revealed a contrast-enhanced mass around the surgical cavity 119.2 months after primary treatment. PET/CT showed high [^68^Ga]Ga-PSMA-11 accumulation in the tumor; with the SUV_max_ 17.4, SUV_mean_ 7.4, TBR 870 and TLR 3.4 (**a**—contrast-enhanced T1WI, **b**—DWI, **c**—PET transverse image, **d**—fused PET/CT transverse image, **e**—maximal intensity projection, **f**—PET sagittal image, **g**—fused PET/CT sagittal image).

**Figure 3:**
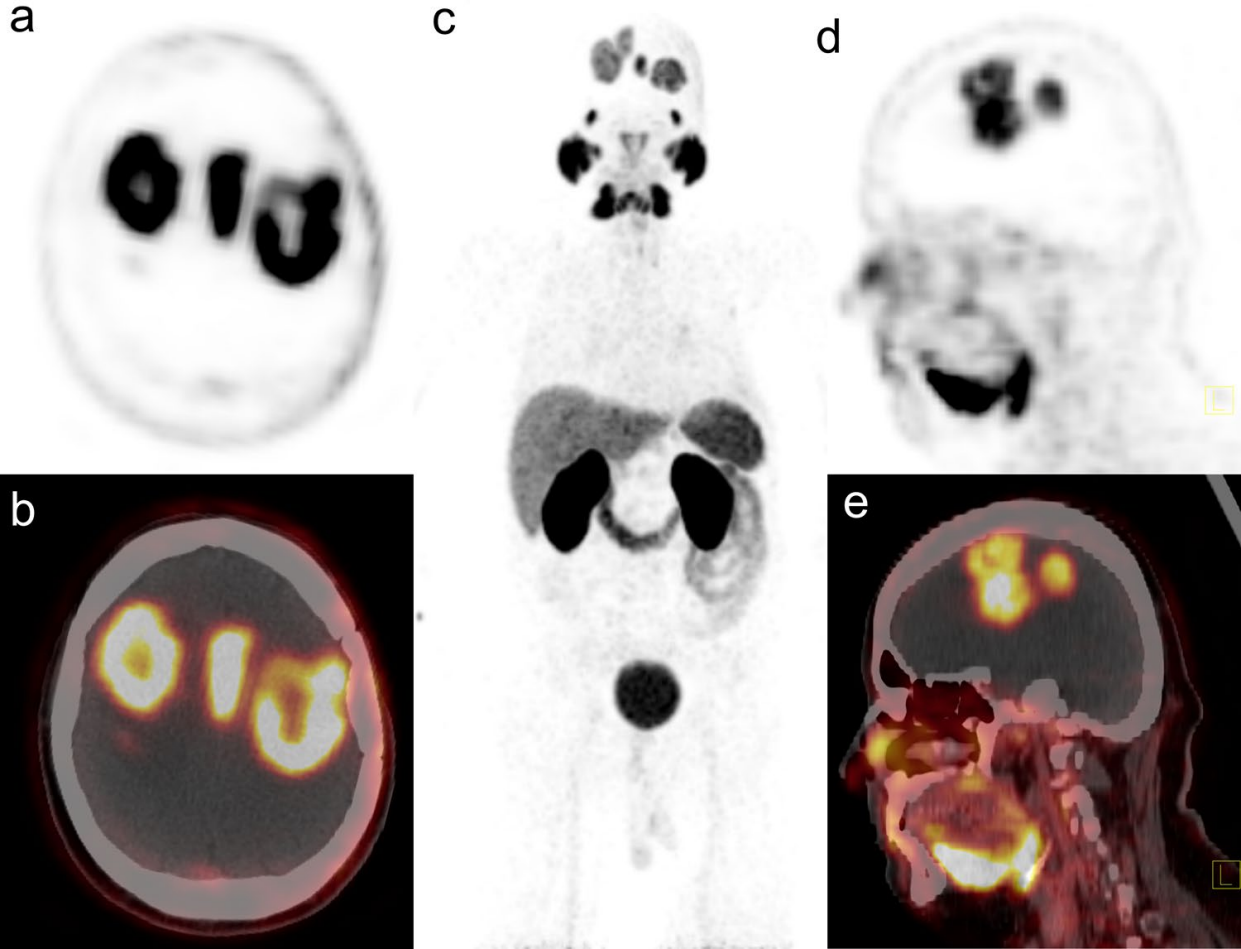
Male, 54 years, with a recurrent multiple high-grade glioma. MR performed 4 months after treatment initiation revealed a mass around the surgical cavity in the left temporo-parietal lobe and multifocal tumors in the right frontal, right parietal lobe and parasagittal area. [^68^Ga]Ga-PSMA-11 PET/CT showed high accumulation in the all tumors with the highest SUV_max_ 15.6, SUV_mean_ 7.5, TBR 156 and TLR 2.8 (**a**—PET transverse image, **b**—fused PET/CT transverse image, **c**—maximal intensity projection, **d**—PET sagittal image, **e**—fused PET/CT sagittal image).

Brain images in different grades of glial tumors are shown in Figs. 1, 2 and 3.

### Correlation between MR and [^68^Ga]Ga-PSMA-11 PET/CT image

All areas with contrast enhancement on MR were associated with increased [^68^Ga]Ga-PSMA-11 accumulation. Subsequently, [^68^Ga]Ga-PSMA-11 PET and MR images were fused using the semiautomatic software with manual adjustment for correct alignment. Visual interpretation showed increased accumulation of [^68^Ga]Ga-PSMA-11 precisely in the contrast-enhanced portions of the tumor at MR. The part of the tumor that was interpreted as necrotic in MR did not show any accumulation of [^68^Ga]Ga-PSMA-11.

### Confirmation of recurrence

Following the PET/CT, the diagnosis of recurrence was confirmed in all patients: by means of histopathology in 16 patients (1–14 days after PET/CT) and by means of follow-up imaging that documented further progression (subsequent MR or another [^68^Ga]Ga-PSMA-11 PET/CT performed 2–6 weeks after PET/CT) in the remaining 18 subjects.

The localization for histopathological sampling was selected in the area of the highest [^68^Ga]Ga-PSMA-11 uptake. We did not record any false-positive scans in comparison to histopathology.

## Discussion

GCP is a transmembrane glycoprotein expressed in the endothelial cells of neovasculature of various tumors (breast and renal carcinoma)^20,21^. From the biochemical point of view, GCP is a zinc-dependent enzyme that plays an important role as a biomarker and a target for the treatment of androgen-resistant prostate carcinoma^22^. Physiologically, GCP is expressed in the membranes of astrocytes and is stored in the presynaptic axonal terminals^23,24^. GCP is coded by the *FOH1* gene located on the short arm of the 11th chromosome at the 11p11.2 position and composed of 19 exons and 18 introns^25^. Due to its first major clinical application in the diagnosis of prostate cancer, in the clinical literature, GCP is frequently referred to as the prostate-specific membrane antigen (PSMA). Based on immunohistochemical examinations, Wernicke et al. reported its expression in all 32 grade IV glioma tumors, specifically in their vasculature^2^. Other authors confirmed a positive result of the PSMA staining in high-grade glioma specimens^8,26^. In contrast, the PSMA expression was relatively infrequent in low-grade gliomas^27^.

These in-vitro results laid the foundation for the new imaging option of glial tumors with radiolabeled PSMA ligands with the use of PET/CT technology. The first case report of [^68^Ga]Ga-PSMA-11 accumulation in glioblastoma multiforme was published in 2015^28^. Later studies showed very promising results on some larger patient series (from 3 to 15 cases) that concordantly reported positive [^68^Ga]Ga-PSMA-11 PET/CT scans in all the examined glioblastoma multiforme cases^10–12,14^. In the present study, the cohort of 34 patients with glial tumors is analyzed. As many as 28 of them had the diagnosis of grade IV glioma, and the results in this subgroup, similarly to the papers mentioned above, demonstrate high tracer uptake in the recurrent tumor. The areas of recurrence presented as foci of intense tracer accumulation with a median SUV_max_ of 7.1 (the highest SUV_max_ value in one of the patients was 15.6—Fig. 3). On the other end of the uptake range, there were some patients with glioblastoma multiforme, with relatively low SUV_max_ (1.3 was the lowest value recorded in this subgroup). However, even these foci of recurrence could be easily delineated at the PET/CT scans because of generally extremely low [^68^Ga]Ga-PSMA-11 uptake in the normal brain parenchyma. The median SUV_max_ of the normal brain was as low as 0.1. Adequately, the contrast between the malignant infiltration and the background was very good in all the grade IV cases. The subsequently high TBR values (median 89.6) are concordant with the in-vitro findings of absent GCP expression in the normal brain vasculature^2,8^. Due to the remarkable TBR, [^68^Ga]Ga-PSMA-11 PET/CT offers an advantage over other tracers. For example, a median TBR of 3.8 was reported recently for [^11^C]methionine PET/MR in newly diagnosed glioblastoma^29^ and median TBR of 3.2 was obtained in [^18^F]fluoroethyltyrosine PET/MR in a group of 32 patients with high-grade glioma recurrence^30^.

Interestingly, we did not record any false-positive scans. A single case of an increased PSMA ligand accumulation in radionecrosis has been published till now^31^. This finding may represent a potential limitation to the application of GCP-targeted agents, but it is not supported by our data.

The representation of cases with glial tumors of other grades than IV seems to be an additional value of our study. Despite the fact that the subgroup of grade III contains only six cases, it can still be considered the largest collective reported so far. Considering that three of these patients had a multifocal recurrence, the per-lesion analysis of 11 tumors in total could be performed. Moreover, the uptake parameters in multiple tumors of the same patient were not always similar. For example, in the patient with four foci of oligodendroglioma relapse (patient No. 4), the SUV_max_ ranged from 0.9 to 11.2. This anecdotal observation provides another evidence of biological heterogeneity of multifocal tumors. In general, performance of [^68^Ga]Ga-PSMA-11 PET/CT in grade III was as excellent as in grade IV. Similarly, high uptake parameters were measured in this subgroup. Some positive experiences with the PSMA imaging in grade III gliomas were also shared by Sasikumar et al., whose two patients with anaplastic oligodendroglioma and anaplastic astrocytoma presented with TBR of 11.9 and 27.0^12^.

Comparison of PET and MR scans showed that the accumulation of [^68^Ga]Ga-PSMA-11 was found precisely in the tumor regions that showed contrast enhancement. Gadolinium-based contrast agents shorten T1 relaxation times and increase tissue contrast by accentuating areas where contrast agents have leaked into the interstitial tissues crossing the blood–brain barrier. The breakdown of the barrier and neoangiogenesis are key features seen not only in primary tumors but also in the recurrences. A better indicator of angiogenesis is the increase in rCBV. In the group of 15 patients for whom this study was available, a clear correlation was found between the accumulation of [^68^Ga]Ga-PSMA-11 and rCBV. On the other hand, the region of T2WI/FLAIR hyperintense signal abnormality surrounding the enhancing part of a tumor or its recurrence is typically referred to as peritumoral edema and can be of vasogenic or infiltrative nature^32^. The infiltrative edema in gliomas represents a mixture of vasogenic edema and infiltrating tumor cells and can be considered as a non-enhancing tumor without pathological angiogenesis and with preserved integrity of the blood–brain barrier. In fact, in many gliomas, the T2WI/FLAIR hyperintense signal abnormality may be indistinguishable from the primary mass lesion^33^. It seems that the processes responsible for both, the imaging patterns in MR and the uptake of [^68^Ga]Ga-PSMA-11, are similar and this explains the obtained results.

The discovery of GCP expression in glial tumors has provided rationale for this particular imaging modality but it can also be considered in the context of the targeted radionuclide therapy with β or α-emitters. First experimental therapy of glioblastoma multiforme using [^177^Lu]Lu-PSMA-617—another targeting ligand has been recently published by our group^34^. Despite earlier concern about a quick washout of the compound due to the non-specific uptake in microvascular endothelium (instead of the tumor cells), we were able to demonstrate a long-lasting accumulation of the β-emitting radioligand within the tumor. This finding seems to predict good chances for the development of this kind of treatment. For the qualification to GCP-targeted radionuclide therapy the TLR of more than 1.5 is commonly used as a qualification criterion to the radionuclide therapy^35^. If the [^68^Ga]Ga-PSMA-11 PET/CT had been used for qualification to the radionuclide therapy in this group, the criterion of TLR > 1.5 would have been fulfilled by one third of the patients.

There are some limitations to this study. First, the sample size in grade III is small, so the differences in the measured parameters between grade III and grade IV subgroups, if any, do not reach statistical significance. Therefore, more extensive research on a larger number of patients, possibly multicenter trials, are warranted. The relatively small number of cases included is the result of strict inclusion criteria: only patients with a suspicion of recurrence were qualified. PET/CT scans obtained at different clinical settings, i.e. for staging or treatment response were not included. This rule should be appreciated as an advantage of this paper. But on the other hand, it could be considered as weakness, since only true-positive scans were acquired. Presence of patients without recurrence, with false-positive findings or with a negative scan despite relapse, would allow us to calculate the sensitivity and specificity.

## Conclusion

In this study, the high GCP expression in the recurrent glioma has been demonstrated in vivo, with the use of [^68^Ga]Ga-PSMA-11 PET/CT. As the treatment options in recurrent glioma are limited, demonstration of GCP expression in the recurrence opens new perspectives for the development of novel radionuclide treatment modalities in brain tumors.

## Abbreviations

GCP: Glutamate carboxypeptidase II
PSMA: Prostate specific membrane antigen
SUV_max_: Maximal standardized uptake value
SUV_mean_: Mean standardized uptake value
TBR: Target-to-background ratio
TLR: Target-to-liver ratio
